# LysoPC and PAF Trigger Arachidonic Acid Release by Divergent Signaling Mechanisms in Monocytes

**DOI:** 10.1155/2011/532145

**Published:** 2011-09-11

**Authors:** Janne Oestvang, Marit W. Anthonsen, Berit Johansen

**Affiliations:** ^1^Department of Biology, Norwegian University of Science and Technology, 7491 Trondheim, Norway; ^2^Faculty of Medicine, Norwegian University of Science and Technology, 7491 Trondheim, Norway; ^3^Section for Biotechnology, Molecular, and Environmental Biology, Department of Biology, Norwegian University of Science and Technology, 7491 Trondheim, Norway

## Abstract

Oxidized low-density lipoproteins (LDLs) play an important role during the development of atherosclerosis characterized by intimal inflammation and macrophage accumulation. A key component of LDL is lysophosphatidylcholine (lysoPC). LysoPC is a strong proinflammatory mediator, and its mechanism is uncertain, but it has been suggested to be mediated via the platelet activating factor (PAF) receptor. Here, we report that PAF triggers a pertussis toxin- (PTX-) sensitive intracellular signaling pathway leading to sequential activation of sPLA_2_, PLD, cPLA_2_, and AA release in human-derived monocytes. In contrast, lysoPC initiates two signaling pathways, one sequentially activating PLD and cPLA_2_, and a second parallel PTX-sensitive pathway activating cPLA_2_ with concomitant activation of sPLA_2_, all leading to AA release. In conclusion, lysoPC and PAF stimulate AA release by divergent pathways suggesting involvement of independent receptors. Elucidation of monocyte lysoPC-specific signaling mechanisms will aid in the development of novel strategies for atherosclerosis prevention, diagnosis, and therapy.

## 1. Introduction

Lysophosphatidylcholine (lysoPC) is found at elevated levels in atherosclerotic lesions [1] and has been postulated to be an important causal agent in inflammation and atherosclerosis. It is a prominent phospholipid component of oxidized low-density lipoproteins (oxLDL), and we have earlier shown that secretory phospholipase A_2_- (sPLA_2_-) modified LDL [2] and lysoPC alone [3] can induce proinflammatory activation of human-derived monocytes by increased release of arachidonic acid (AA). LysoPC may be responsible for various cellular processes such as regulation of monocyte adhesion molecule expression [4], chemoattractant properties [5], and monocyte proinflammatory cytokine secretion [6]. Some of the intracellular signaling events initiated by lysoPC are the activation of phospholipase D (PLD) [7, 8] and stimulation of p38 and p42/44 mitogen-activated protein kinases (MAPKs) through G_i_/G_o_ proteins [9]. Nevertheless, the signaling mechanisms regulating specific cellular processes of lysoPC are not completely understood.

It has been argued that lysoPC triggers cellular signaling through G-protein-coupled receptors. To our knowledge, three different receptors have been suggested as lysoPC responsive receptors. First, evidence was presented showing that lysoPC initiates intracellular signaling through the platelet-activating factor (PAF) receptor [6, 8, 10], and it was hypothesized that both lysoPC and PAF-induced common signaling pathways through the PAF receptor. Later, two new G-protein-coupled receptors specific to lysoPC, G2A and GPR4, were described [11, 12]. However, the data showing direct binding of lysoPC to these receptors have been retracted due to their irreproducibility [13]. In spite of this, there is still evidence of a functional relationship between lysoPC and the G2A receptor [14–17]. In addition, lysoPC has been reported to activate G_*α*s_-proteins and induce apoptosis through the G2A receptor in primary lymphocytes [17], which indicates that the G2A receptor is a G_*α*s_-receptor. Hence, there is still uncertainty if and how lysoPC induces intracellular signaling.

PAF plays important roles in inflammation-mediating cell-cell interactions in models of acute and chronic inflammation in virtually all organs and induces cellular responses through the G-protein-coupled PAF receptor [18]. Compared to lysoPC, the intracellular signaling of PAF has been extensively studied, and PAF is known to activate both PLD and p38 MAPK through its receptor [19–22]. Additionally, a protein kinase C-dependent PAF-induced pathway that releases AA has been suggested [23]. PAF-induced cPLA_2_ activity is inhibited by pertussis toxin (PTX), suggesting G_*α*i_-protein involvement [24].

Enzymes that can mediate AA release are the PLA_2_ enzymes that hydrolyze fatty acids from the sn-2 position in membrane phospholipids. This results in the generation of biologically active lipids, such as free fatty acids and lysophospholipids, all of which regulate inflammation. PLA_2_ enzymes comprise a large family that is diverse in structure, biological function, mechanism, localization, and regulation, and the isoenzymes are classified thereby [25]. cPLA_2_ is preferentially arachidonyl selective and requires submicromolar amounts of Ca^2+^ for activity [26, 27]. In a variety of cell types, phosphorylation and activation of cPLA_2_ is brought about by MAPK [28–30]. Extracellular PLA_2_, also referred to as sPLA_2_, is secreted by cells in response to inflammatory stimuli, and is thought to augment the inflammatory process by catalyzing the production of lipid mediators. sPLA_2_ has been associated with many physiological and pathophysiological processes such as rheumatoid arthritis, sepsis, psoriasis, and atherosclerosis [31–33]. 

In order to examine if lysoPC intracellular signaling is induced by an independent G-protein-coupled receptor, we compared lysoPC- and PAF-induced intracellular signaling components leading to AA release. Our results suggest that PAF triggers a PTX-sensitive pathway leading to sequential activation of sPLA_2_, PLD, and cPLA_2_ and AA release. In contrast, lysoPC initiates two pathways, where one sequentially activates PLD and cPLA_2_, and a second PTX-sensitive pathway that activates cPLA_2_ with the simultaneous activation of sPLA_2_, all leading to AA release initiated from independent receptors. In conclusion, our results suggest that lysoPC indeed induces AA release via different signaling pathways compared to PAF. This supports the hypothesis of divergent signalling pathways for the two lipids based on their binding to unique receptors.

## 2. Materials and Methods

### 2.1. Materials

RPMI 1640, gentamicin, lysoPC C:16, 2-butanol, 3-(4,5-dimethylthiazol-2-yl)-2,5-difenyl tetrazolium bromide (MTT), PTX, Triton-X100, EDTA, EGTA, PMSF, and fatty acid-free bovine serum albumin (BSA) were purchased from Sigma Chemical Co. (St. Louis, USA). Fetal calf serum was obtained from Integro b.v. (Holland) and WEB2170 from Boehringer Ingelheim (Germany). [^3^H]AA and [^14^C]-L-3 phosphatidylcholine, 1 stearoyl-2-arachidonyl were purchased from NEN (Boston, USA) and methyl[^14^C]choline from Amersham LIFE SCIENCE (Buckinghamshire, England). Biologically active PAF C:16 and methyl arachidonyl fluorophosphonate (MAFP) were purchased from Cayman Chemical (USA). NaOH, methanol, HCl, NaCl, NH_4_OH, 1-butanol, aluminium sheets silica gel 60 TLC plates, and chloroform were purchased from Merck (Darmstadt, Germany). L-glutamine was obtained from Gibco BRL (Life Technologies, Grand Island, NY, USA). Leupeptin and pepstatin were obtained from Roche Diagnostics GmbH (Mannheim, Germany) and Bio-Rad reagent was from Bio-Rad Laboratories (Hercules, USA). Group IIA sPLA_2_ inhibitor, SB203347, was generously donated by James Winkler, Smith Kline Beecham (Pharmaceuticals, Pa, USA).

### 2.2. Cell Culture

THP-1 cells were maintained in suspension for passage and growth in RPMI 1640 containing 10% (v/v) fetal calf serum, 3 mg/mL glutamine, and 0.1 mg/mL gentamicin. The cells were grown with an initial density of 2 × 10^5^ cells/mL and subcultured 2 times a week to ensure continuous logarithmic growth in a humidified 5% v/v CO_2_ atmosphere. The THP-1 monocytes were when indicated differentiated with 160 nM PMA for 24 h. Differentiation from monocytes to macrophages was monitored by changes in morphology and adherent capacity. The THP-1 cell line was bought from ATCC and regularly checked for mycoplasma contamination. 

### 2.3. Measurement of Extracellular [^3^H] AA Release

THP-1 cells at 3 × 10^5^ cells/well were labeled with [^3^H]AA (0,3 *μ*Ci/mL) and starved in 0.5% v/v FCS RPMI 1640 for 16 h as previously described [33]. Extracellular release of total [^3^H] was measured by scintillation counting after stimulation with lysoPC or PAF for indicated concentrations and time periods in RPMI 1640 containing 1 mg/mL BSA. In experiments using antagonists and inhibitors, WEB2170 was incubated for 15 min, alcohols and MAFP for 30 min, SB203347 for 1 h, and PTX for 2 h prior to stimulation. Results are given as released [^3^H]AA in the supernatant relative to [^3^H]AA incorporated in the cells and are normalized to show fold induction of treated relative to untreated cells. In experiments with inhibitors, results are shown as percent inhibition of lysoPC of PAF-induced cells. Results shown are representative of three independent experiments. 

### 2.4. RNA Isolation

Total cellular RNA was isolated by TRIzol extraction according to the manufacturer's instructions (Gibco BRL, Life Technologies Inc., Grand Island, NY, USA). Briefly, cells were lysed and homogenized using Trizol reagent. Chloroform was added before centrifuging for phase separation. The aqueous phase with RNA was transferred to a new tube, and RNA was precipitated by mixing the aqueous phase with isopropyl alcohol. RNA was pelleted by centrifuging and washed with ethanol before it was air-dried and suspended in DEPC-treated water. RNA concentration was determined by spectrophotometry at A_260_.

### 2.5. RT-PCR Detection of Different PLD Isoforms

Total RNA was reverse-transcribed and amplified by PCR as previously described [3]. Conditions for RT-PCR were hPLD1a and hPLD1b, 94°C for 30 sec, 60°C for 1 min, and 72°C for 1 min for 27 cycles and for hPLD2, 94°C for 1 min, 56°C for 1.5 min and 72°C for 2 min for 30 cycles. hPLD1a and hPLD1b are transcript variants of the same gene and primers were designed to distinguish the two transcript variants, which results in products of 638 bp (hPLD1a) and 533 bp (hPLD1b) [34]. Primers used for amplification were synthesized as follows: hPLD1a and hPLD1b fwd 5′-TGGGCTCACCATGAGAA-3′ (nucleotides 1475–1491) and rew 5′-GTCATGCCAGGGCATCCGGGG-3′ (nucleotides 2133–2113) and hPLD2 fwd 5′-TCCATCCAGGCCATTCTGCAC-3′ (nucleotides 2802–2778) and rew 5′-CTATGTCCACATTTCTAGGGGGAT-3′ (nucleotides 2802–2778). All PCR reactions were performed with water as the negative control both for the RT reaction and PCR reaction (not shown).

### 2.6. PLD Choline Assay

THP-1 cells were starved in RPMI 1640 supplemented with 1 mg/mL BSA and [^14^C]choline (0.6 *μ*Ci/mL) for 40 h with a cell density of 6.75 × 10^5^ cells/well. The cells were stimulated with lysoPC (40 *μ*M, 2 min) or PAF (40 *μ*M, 2 min) in RPMI 1640 with 1 mg/mL BSA. Preincubations with inhibitors were conducted under same conditions as for AA-release. To extract aqueous phase metabolites the medium was centrifuged to remove cells before ice-cold chloroform/methanol (1 : 2, v/v) was added. To the medium-chloroform/methanol solution, ice-cold chloroform and water was added to a final ratio of methanol/chloroform/water (2 : 2 : 1, v/v/v). The solution was mixed well and centrifuged (500 g, 5 min) to separate the organic phase from the water phase. The upper aqueous phase was evaporated to dryness in a vacuum dryer overnight and resolved in water/ethanol (1 : 1, v/v). Free choline was separated from phosphocholine by TLC on aluminum silica gel 60 sheet developed with 0.9% w/v NaCl/methanol/NH_4_OH (50 : 50 : 5,v/v/v). Released choline in the medium was measured by Phospho-Imager. 1 M NaOH were added to the cells and the radioactivity was measured by liquid scintillation counting to determine total radioactivity in cells. Results are expressed as released choline in the medium relative to total radioactivity and are normalized to show fold induction of treated relative to untreated cells. In experiments with inhibitors, results are shown as percent inhibition of lysoPC or PAF-induced cells. Percent inhibition is calculated with data retracted from the background noise. Results given are representative of three independent experiments.

### 2.7. cPLA_2_ In Vitro Assay

cPLA_2_ activity was measured as described previously [35]. In brief, THP-1 cells were seeded (2 × 10^6^ cells/well) and starved (0,5% v/v FCS, 18 h) before stimulation with lysoPC (40 *μ*M, 10 min) or PAF (40 *μ*M, 5 min). Preincubations with inhibitors were done under the same conditions as for AA-release. The cells were lysed in lysis buffer and protein concentrations were measured by BioRad protein reagent. 100 *μ*g lysate was incubated with [^14^C]-L-3 phosphatidylcholine, 1 steroyl-2-arachidonyl-micelles (100 *μ*M) at 37° centigrade for 30 min before lipid extraction by the Bligh and Dyer method [36]. Free AA was separated from other phospholipids by TLC on aluminum silica gel 60 sheets developed with ethyl acetate/isooctane/acetic acid/water (55/75/8/100, v/v/v/v). The amount of produced AA was detected by Phosphor-Imager and the cPLA2 activity was expressed as a percentage relative to total amount of phospholipids. In experiments with inhibitors, results are shown as percent inhibition of lysoPC or PAF-induced cells. Percent inhibition is calculated with data retracted from the background noise. Results shown are representative of three independent experiments.

### 2.8. MTT Cytotoxicity Assay

LysoPC and different chemical inhibitors were tested for cytotoxicity by MTT assay as previously described [37]. The MTT assay reflects the mitochondrial dehydrogenase activity, and the absorbance at 580 nm was used as an index of cell viability.

### 2.9. Statistics

The data are shown as means ± SD of separate experiments each containing 3 parallels (arachidonate and PLD assay) or 2 parallels (cPLA2 assay). For the arachidonate and PLD assay, the data of three independent experiments were compared with the Kruskal-Wallis test for random samples, and those at *P* < 0.05 were considered significant. Each set of experiments was repeated three times.

## 3. Results

### 3.1. LysoPC and PAF Stimulate [^3^H]AA Release in THP-1 Cells

**Figure 1:**
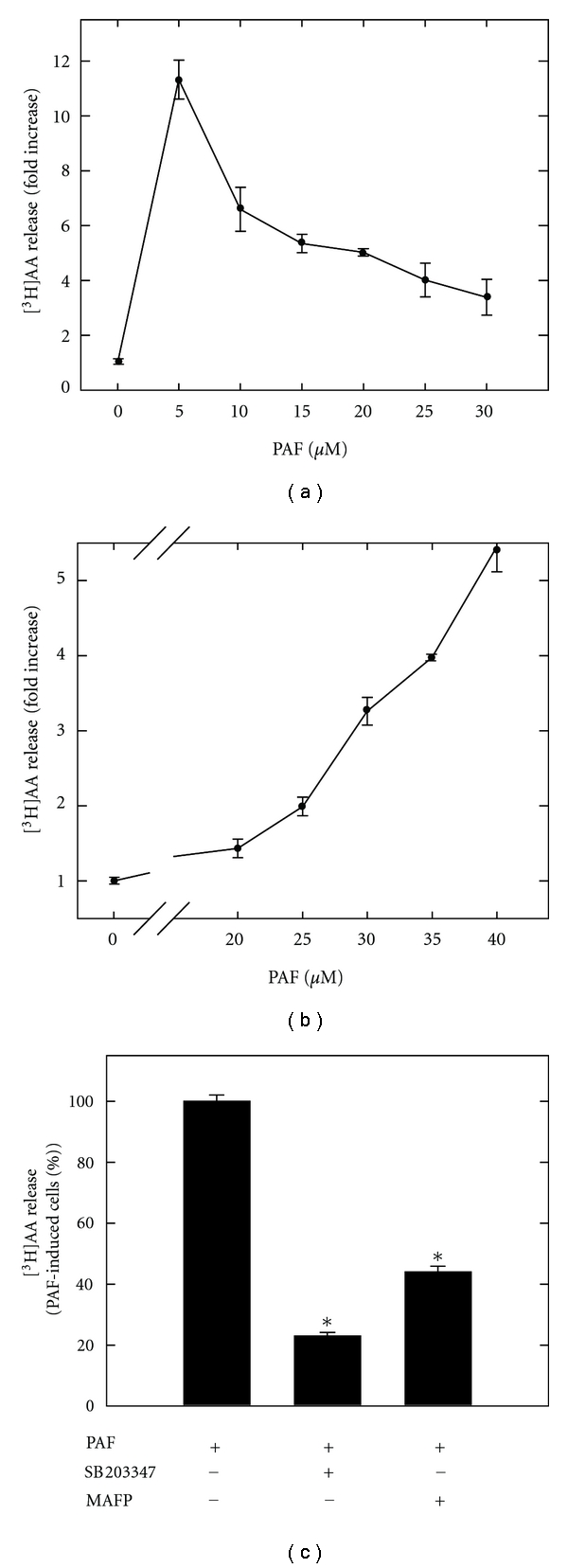
PAF and [^3^H]AA release in human monocytes. Prelabeled THP-1 cells were stimulated with PAF, and [^3^H]AA release was measured by liquid scintillation counting. (a) The time course of PAF- (35 *μ*M) mediated [^3^H]AA. (b) PAF stimulation for 5 min mediates [^3^H]AA release in a dose-dependent matter. (c) Shows inhibition of PAF-induced [^3^H]AA by the sPLA_2_ inhibitor SB203347 (10 *μ*M) and the cPLA_2_ inhibitor MAFP (10 *μ*M). The PAF concentration used is 35 *μ*M. Data are expressed as means ± SD of triplicate determinations within separate experiments. Asterisks indicate that values are statistically different from PAF-treated cells (∗).

We have earlier shown that lysoPC stimulates [^3^H]AA and [^14^C]OA release in the human-derived monocytic cell line, THP-1, mediated both by sPLA_2_ and cPLA_2_ [3]. In order to achieve a more detailed understanding of the mechanism of the lysoPC-induced pathway and, additionally, to distinguish it from pathways induced by other lysolipid analogues, we tested analogues such as lysophosphatidic acid, sphingosylphosphorylcholine (conc. ranging from 20 to 100 *μ*M and stimulation time varying from 10 to 120 min, results not shown), and PAF for their ability to trigger AA release. Among the analogues tested, only PAF could induce significant AA release (Figure 1). PAF-stimulated [^3^H]AA release in a dose- and time-dependent manner with a maximal release after five minutes (Figure 1(a)) at an optimal concentration of 35 *μ*M PAF (Figure 1(b)). Comparatively, lysoPC stimulated AA release with a maximum after ten minutes at an optimal concentration of 40 *μ*M [3]. Hence, we observed that PAF elicits AA release with slightly more rapid kinetics compared to lysoPC. 

It is difficult to define PAF's “physiological concentration”, but the optimal concentrations of PAF and lysoPC were selected based on the criteria that the cells were viable, as measured by MTT assay [38–40]. To enhance the sensitivity of the AA assay, fatty acid-free BSA was added to the media. Hence, the final concentrations of free lipid in the stimulation media were less than the actual concentration used [41]. By stimulating THP-1 monocytes with 40 *μ*M lysoPC for 10 min or 35 *μ*M PAF for 5 min, we did get a fold increase in AA release between 3 to 12.

In order to determine which PLA_2_ enzymes contribute to AA release, different PLA_2_ inhibitors were applied. The sPLA_2_ inhibitor, SB203347, reduced PAF-stimulated AA release by 80% v/v (Figure 1(c)), while the cPLA_2_/iPLA_2_ inhibitor, MAFP, reduced AA release by 60% v/v (Figure 1(c)). Consequently, our results suggest that PAF stimulate cPLA_2_ or iPLA_2_ activity; hence, the cPLA_2_ activity assay is used later in this study to further assess cPLA_2_ activation. Our results suggest that PAF-stimulated AA release is mediated by sPLA_2_ and cPLA_2_ or iPLA_2_ in THP-1 cells, similar to the lysoPC-triggered response.

### 3.2. LysoPC and PAF Stimulate PLD Activity by Independent Mechanisms

**Figure 2:**
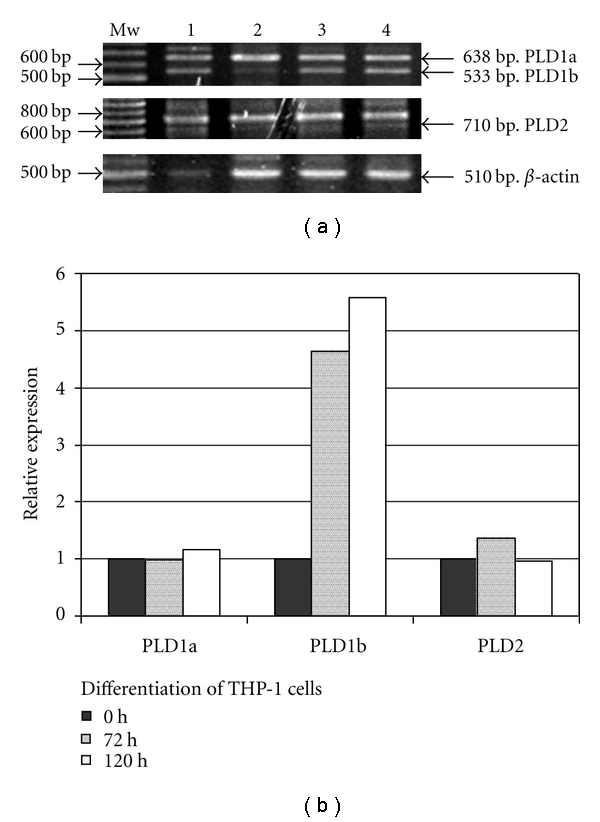
Expression of PLD isoforms in THP-1 cells. RT-PCR with primers specific for different PLD isoforms (PLD1a 638 bp, PLD1b 533 bp, and PLD2 710 bp) was performed on total RNA isolated from undifferentiated THP-1, THP-1 cells differentiated for 72 h and 120 h (lanes 2,3, and 4, resp.; lane 1 is the positive control). Band intensities were calculated using BioRad image analysis software and fold induction of PLD mRNAs (normalized to b-actin) relative to undifferentiated cells is shown in the lower panel.

It has been shown that lysoPC [8] and PAF [19] stimulate PLD activity in mouse peritoneal macrophages. PLD is a widely expressed enzyme, of which there are three mammalian isoforms, PLD1a, PLD1b, and PLD2 [42]; all three isoenzymes are expressed in human monocytes [43]. To our knowledge, the expression of PLD isoenzymes in THP-1 cells has not been analyzed. To assess which PLD enzymes were expressed, the PLD mRNA expression pattern was analyzed by RT-PCR. We did indeed find expression of PLD1a and PLD2 in both undifferentiated and differentiated THP-1 cells, while PLD1b was faintly expressed in undifferentiated cells and clearly upregulated in differentiated THP-1 cells (Figure 2). Since we use undifferentiated THP-1 cells in this study, any PLD activity we measure would be due mainly to PLD1a and/or PLD2. 

**Figure 3:**
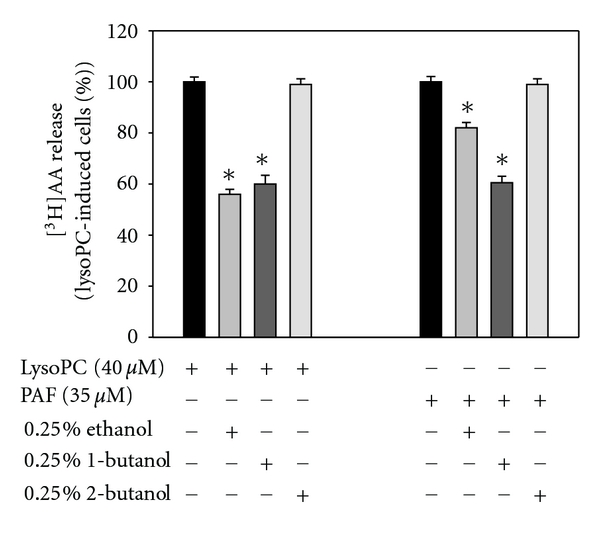
LysoPC- and PAF-mediated [^3^H]AA release is inhibited by different alcohols. Prelabeled THP-1 cells were preincubated with different alcohols 30 min before challenge with lysoPC (40 *μ*M, 10 min) or PAF (35 *μ*M, 5 min). [^3^H]AA release in the medium was measured by liquid scintillation counting. Data are expressed as means ± SD of triplicate determinations within separate experiments. Asterisks indicate that values are statistically different from PAF- or lysoPC-treated cells (∗).

In order to determine if PLD is involved in lysoPC- and PAF-stimulated AA release in human-derived monocytes, [^3^H]AA prelabeled THP-1 cells were preincubated with the primary alcohols 1-butanol and ethanol and with the secondary alcohol 2-butanol. The primary alcohols, but not the secondary alcohol 2-butanol, will attenuate signaling mediated by PLD, as they compete with water to be the hydroxyl donor in the hydrolysis of phospholipids by PLD. [^3^H]AA release in response to lysoPC and PAF stimulation was partially inhibited by the two primary alcohols, 1-butanol and ethanol (Figure 3) although ethanol was less effective in inhibiting [^3^H]AA release by PAF than by lysoPC. As expected, the secondary alcohol, 2-butanol, did not inhibit [^3^H]AA release (Figure 3) because of its inability to be a hydroxyl donor. This suggests that the enzymatic product of PLD activity, phosphatidic acid (PA), regulates lysoPC- and/or PAF-stimulated AA release in THP-1 cells.

**Figure 4:**
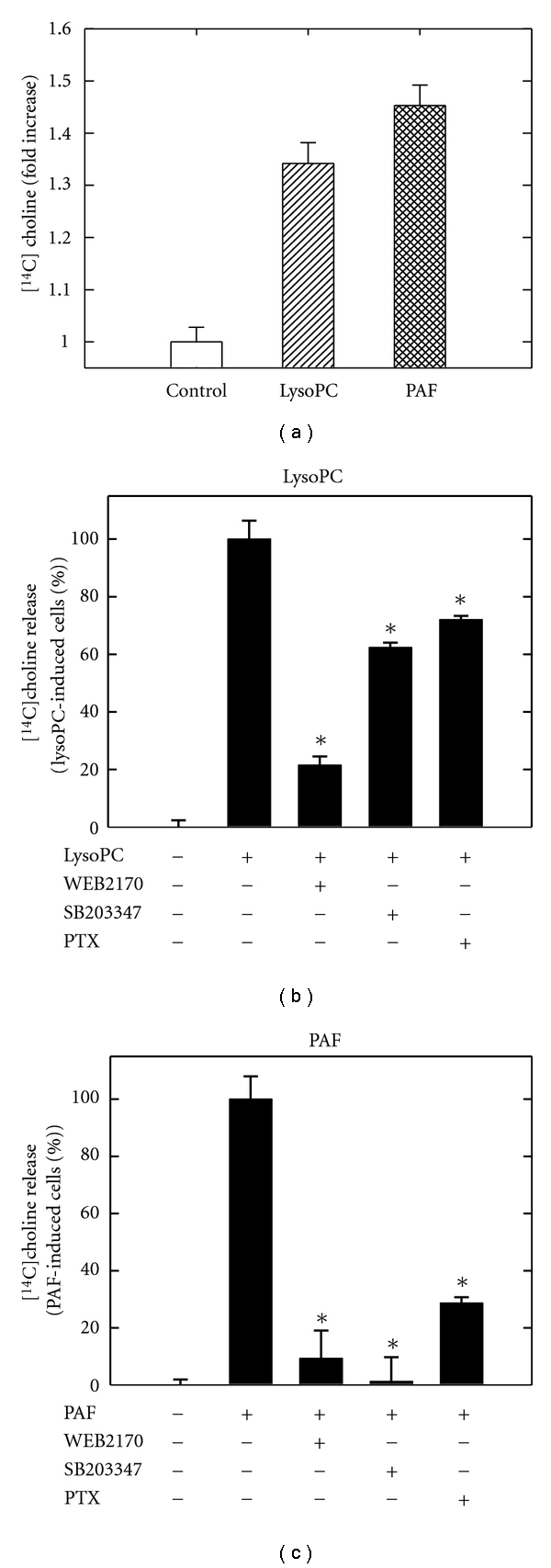
LysoPC and PAF stimulate PLD activity. (a) [^14^C]choline prelabeled THP-1 cells were stimulated with lysoPC or PAF for two minutes, and [^14^C]choline released in the medium was measured after TLC separation. (b) and (c) [^14^C]choline prelabeled THP-1 cells were preincubated with WEB2170, SB203347, or PTX before (b) lysoPC or (c) PAF challenge. Data are expressed as means ± SD of triplicate determinations within separate experiments. Asterisks indicate that values are statistically different from PAF- or lysoPC-treated cells (∗).

To further assess if PLD is activated by lysoPC or PAF, PLD activity was analyzed by cellular release of [^14^C]choline. Both lysoPC and PAF significantly increased [^14^C]choline release, which is an indication of PLD activity (Figure 4(a)). Optimal release was at 40 *μ*M after 2 minutes for both lysoPC and PAF. This suggests that both lysoPC and PAF stimulate PLD activity. 

PAF receptor is a G-protein coupled receptor and lysoPC is also believed to mediate intracellular signaling through G-protein-coupled receptors. To determine if different G-protein coupled receptors were involved in PLD activation, we applied different inhibitors to the cells before stimulation of PLD activity. The PAF antagonist WEB2170 (10 *μ*M) inhibited [^14^C]choline release in response to lysoPC by 80% (Figure 4(b)) and in response to PAF by 90% (Figure 4(c)). The PAF receptor is regulated by G_*α*i_-proteins [24]. The G_*α*i_-protein inhibitor PTX inhibited lysoPC-induced PLD activation by about 30% (Figure 4(b)) and PAF-induced PLD activation by 70% (Figure 4(c)), suggesting that a G_*α*i_-protein is more central in the PAF-initiated stimulation of PLD compared to lysoPC.

Since sPLA_2_ involvement in the lysoPC and PAF signaling pathway is already suggested, we wanted to examine if sPLA_2_ mediates PLD activity. In order to determine if sPLA2 activates PLD, the specific sPLA_2_ inhibitor SB203347 was applied in the PLD experiment. SB203347 inhibited PAF-induced PLD activation by almost 100% (Figure 4(c)), while lysoPC-induced PLD activation was inhibited only by 30% (Figure 4(b)). This suggests that the PAF-induced pathway requires sPLA_2_ for activation of PLD, while in the lysoPC pathway, sPLA_2_ contributes poorly to PLD activation, which is in accordance with a study in HEK293 cells [44]. Additionally, involvement of G_*α*i_-proteins is more prominent in PAF-stimulated PLD activation compared to lysoPC-stimulated activation. Taken together, this suggests that divergent intracellular signaling pathways are initiated by lysoPC and PAF.

### 3.3. cPLA_2_ Activity Is Distinctively Stimulated in Response to LysoPC and PAF

**Figure 5:**
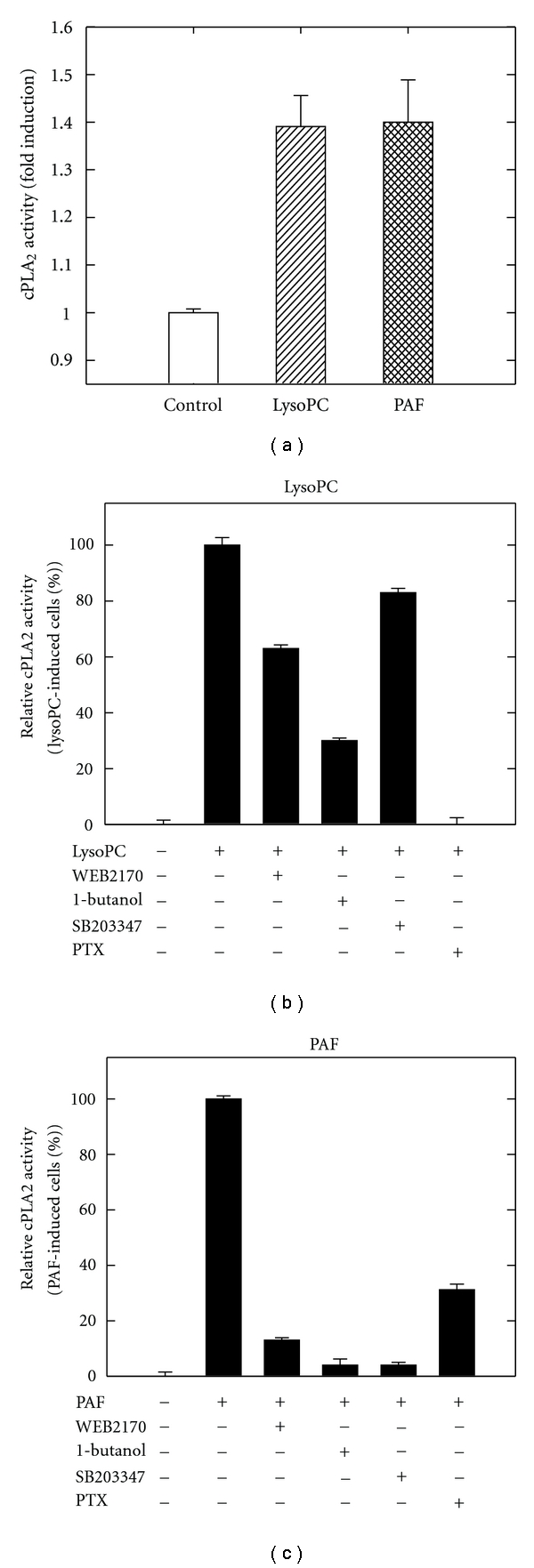
cPLA2 activity is differentially regulated by lysoPC and PAF. (a) cPLA_2_ enzyme assays were done on cell lysates treated with lysoPC or PAF. (b) and (c) cPLA_2_ activity was determined in lysates from THP-1 monocytes treated with different chemical inhibitors before stimulation with (b) lysoPC or (c) PAF. Data are expressed as means ± SD of duplicate determinations within separate experiments. Data shown are one representative of three independent experiments.

We have earlier shown that lysoPC stimulates cPLA_2_ activity in human-derived monocytes [3] and that cPLA_2_ is involved in PAF-mediated AA release by inhibition with MAFP (Figure 1(c)). We examined cPLA_2_ activation in an in vitro assay, and our results showed that cPLA_2_ activity was significantly induced by both lysoPC and PAF (Figure 5(a)). LysoPC-induced responses are inhibited by PAF-receptor antagonists [6, 8, 10], and it has been argued that lysoPC might act through the PAF receptor. To investigate if lysoPC-stimulated cPLA_2_ activity is induced by PAF receptor, we applied the antagonist WEB2170 to the cells prior to analysis of cPLA_2_ activity. LysoPC- and PAF-stimulated cPLA_2_ activity was inhibited by WEB2170 by 40% (Figure 5(b)) and 95% (Figure 5(c)), respectively. This indicates that lysoPC acts partly through PAF receptor and partly thought another unidentified receptor. 

Both lysoPC and PAF are reported to activate PLD in mouse peritoneal macrophages [8, 45]. In Figure 4(a), we show that PLD is activated in human-derived monocytes by lysoPC or PAF. To analyze if PLD was involved in the pathway activating cPLA_2_, we applied 1-butanol to the cells before analysis of cPLA_2_ activity. As shown by the cPLA_2_ in vitro activity assay, LysoPC- or PAF-induced cPLA_2_ activity was indeed inhibited by 1-butanol (0.25% v/v) by 70% (Figure 5(a)) and 95% (Figure 5(b)), which strongly suggests that PLD is involved in cPLA_2_ activation although at a significantly higher degree in response to PAF compared to lysoPC stimulation. Again this suggests distinct signaling for PAF and lysoPC. 

We previously reported that sPLA_2_ contributes to lysoPC-mediated AA release [3], and above, we have shown for the first time that sPLA_2_ also can contribute to PAF-mediated AA release (Figure 1(c)). To analyze if sPLA_2_ regulates lysoPC- or PAF-induced cPLA_2_ activity, we applied the sPLA_2_ inhibitor SB203347. SB203347 inhibited PAF-stimulated cPLA_2_ activity by 95% (Figure 5(c)), while lysoPC-induced stimulation was dramatically less sensitive to the inhibitor (15% reduction, Figure 5(b)). These results indicate that PAF-mediated cPLA_2_ activation is regulated by sPLA_2_; however, lysoPC-mediated cPLA_2_ activity is poorly regulated by sPLA_2_.

LysoPC has been reported to mediate signaling pathways through G_*α*s_-proteins [17]. In this study, we have used PTX, which is a G_*α*i_-protein inhibitor, in order to examine if G_*α*i_-proteins are involved in any of the pathways. PTX inhibited potently both lysoPC-(Figure 5(b)) and PAF-(Figure 5(c)) induced cPLA_2_ activation by 100% and 68%, respectively, suggesting that cPLA_2_ activity induced by lysoPC is regulated through G_*α*i_-proteins and to a lesser extent in the PAF-initiated pathway.

### 3.4. LysoPC and PAF-Stimulated [^3^H]AA Release Is Partly Blocked by the PAF Receptor Antagonist WEB2170

**Figure 6:**
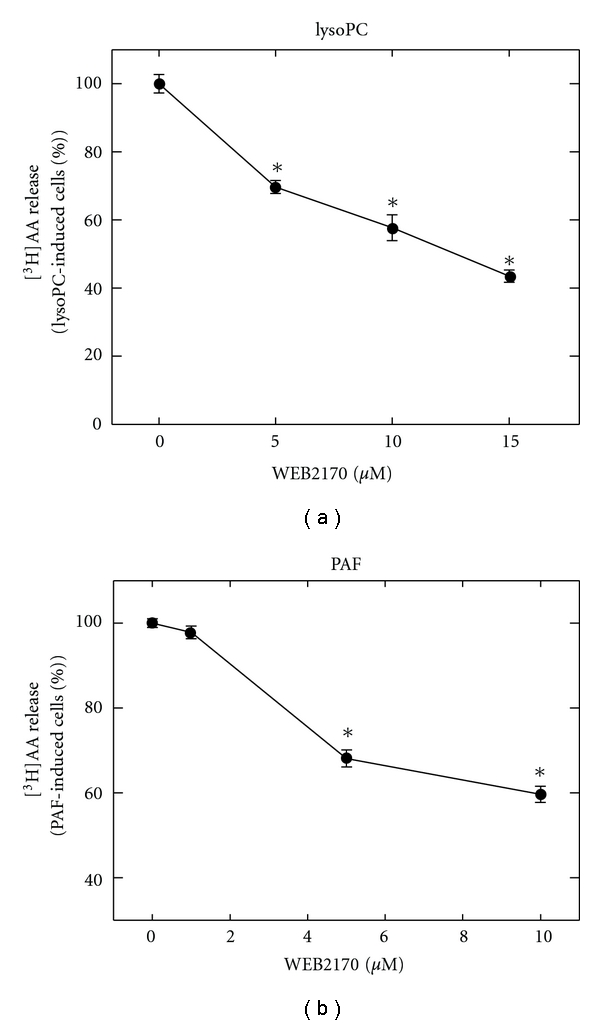
LysoPC-mediated [^3^H]AA release is inhibited by the PAF-receptor antagonist WEB2170. Prelabeled THP-1 cells were preincubated with WEB2170, stimulated with (a) lysoPC (40 *μ*M, 10 min.) or (b) PAF (35 *μ*M, 5 min) and [^3^H]AA release in the medium was measured by liquid scintillation counting. Data are expressed as means ± SD of triplicate determinations within separate experiments. Asterisks indicate that values are statistically different from PAF- or lysoPC-treated cells (∗).

It has been reported that lysoPC may stimulate cells via the PAF receptor [6, 8, 10]. We compared the ability of the PAF receptor antagonist WEB2170 to inhibit AA release by lysoPC and PAF. WEB2170 inhibited both lysoPC- and PAF-stimulated [^3^H]AA release by 40% (Figures 6(a) and 6(b)) at 10 *μ*M WEB2170, suggesting that lysoPC acts partly through PAF receptor and partly through another unidentified receptor. This is similar to what is observed in HL-60 cells [46], regarding lysoPC and PAF's ability to initiate independent signaling mechanisms.

## 4. Discussion

**Figure 7:**
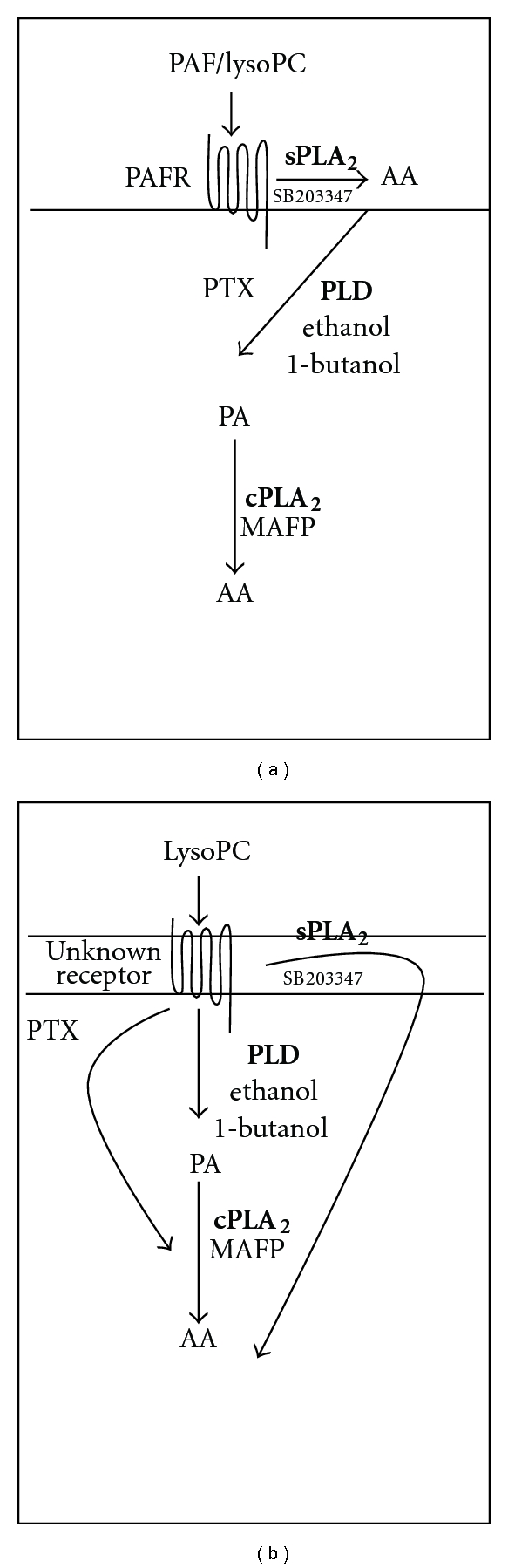
Proposed main molecular mechanisms of intracellular signaling mediated by (a) PAF receptor and (b) unidentified lysoPC-sensitive receptor. The figure shows enzymes in bold/italic and inhibitors in small letters with the sign “–|”.

Taken together, our results suggest that lysoPC and PAF differ in the way they stimulate PLD and cPLA_2_ activation. PAF stimulates PLD and cPLA_2_ activity in a sequential manner mediated by sPLA_2_. In contrast, lysoPC sPLA_2_-induced AA release appears to be in a very low extent dependent of PLD. Moreover, cPLA_2_ is to a large extent regulated by G_*α*i_-proteins in the lysoPC pathway but to a lesser degree in the PAF pathway (as shown in Figure 7).

PLD is reported to be activated in response to lysoPC and PAF [10–12, 47]; however, PLD has, to our knowledge, not been shown to be involved in lysoPC- or PAF-induced AA release. We have shown here that there is an important relationship between PLD, cPLA_2_, and consequently AA release. In addition, there is a striking difference upstream of PLD in the two pathways leading to AA release, where sPLA2 mediates PLD activation in response to PAF but partly in response to lysoPC. These results suggest two divergent pathways for lysoPC and PAF that earlier have been argued to share common signaling pathways. 

Several possible mechanisms have been suggested in the literature about how PLD may affect AA release. Firstly, PLD may activate MAPK cascade that may contribute to phosphorylation of cPLA_2_ and consequently activation of the enzyme [28, 48]. We are currently investigating if p38 may be an intermediate step between PLD and lysoPC induced AA release. Secondly, PLD generates PA, which may be a substrate for PLA_2_ enzymes [49]. Third, sequential activation of PLD and phosphatidate phosphohydrolase results in DAG accumulation. This facilitates the interaction of cPLA2 with its substrate [50]. Thus, our results indicate that PLD is not only important in generation of the key mediator PA but also in activation of other enzymes such as cPLA_2_. Taken together, PLD seems to be important in order to differentiate between the lysoPC- and PAF-induced intracellular signaling pathways.

Previously, both lysoPC and PAF have been shown to release AA in human-derived monocytes [3, 45]. Interestingly, our results suggest that the AA release is a result of two independent pathways, one initiated by PAF and a different pathway initiated by lysoPC. This novelty underscores lysoPC and PAF's ability to initiate and contribute to inflammation through the activation of PLA_2_ enzymes and the consequent release of AA, which is a precursor for the proinflammatory hormones, the eicosanoids. Moreover, the two distinct pathways triggered by the two lipids suggest that AA release can be regulated by divergent pathways, mediating similar proinflammatory response.

PAF-stimulated AA release has in cell systems other than monocytes been shown to be inhibited by the G_*α*i_-protein inhibitor, PTX, indicating that G_*α*i_-proteins are involved in PLA_2_ activation [51]. It is known that lysoPC can mediate cellular responses through several G-proteins ([17] and references herein). In our study, the PAF-mediated AA releasing pathway was clearly regulated by a G_*α*i_-protein. In contrast, G_*α*i_-proteins were important for cPLA_2_ activation but not as much in regulating PLD activity in response to lysoPC. Thus, indicating a third pathway for AA release by lysoPC. Hence, our results suggest that the PAF pathway and in part the lysoPC pathway is triggered by G_*α*i_-protein coupled receptors.

It is well known that PAF induces intracellular signaling through the G-protein coupled PAF receptor and that it is inhibited by different PAF antagonists such as WEB2170 [52]. In addition, it is reported that lysoPC-induced responses may be inhibited by PAF-receptor antagonists [6, 8, 10], and previous authors suggest that this indicates cellular lysoPC-induced responses via the PAF receptor. We show here that both lysoPC- and PAF-stimulated cellular responses were inhibited by the PAF receptor antagonist, WEB2170 although the intracellular signaling pathways inhibited clearly were different. The literature opens for the existence of structurally related receptors effectuating lysoPC- or PAF-stimulated responses, and further studies will be necessary to identify a specific receptor for lysoPC. Hence, our results strongly suggest that lysoPC and PAF stimulate independent signaling pathways leading to AA release are partly triggered by independent receptors, but we are uncertain as to the identity of the exact receptor responsible for the major lysoPC-induced intracellular signaling.

In this study we come closer to resolving some of the molecular mechanisms regulating AA release in response to lysoPC in human-derived monocytes by comparing it to that of its analogue PAF. In conclusion, our results suggest separate pathways leading to AA release stimulated by PAF compared to lysoPC (Figure 7). PAF triggers a PTX sensitive pathway by sequentially activating sPLA_2_, PLD, and cPLA_2_, while lysoPC triggers one pathway by sequentially activating PLD and cPLA_2_, a second PTX sensitive pathway activating cPLA_2_ and third pathway activating sPLA_2_. More detailed understanding of intracellular signaling mechanisms will allow for greater specificity in the design of future therapeutic strategies.

## Abbreviations

lysoPC: Lysophosphatidylcholine
PLA_2_: Phospholipase A_2_
PAF: Platelet activating factor
PLD: Phospholipase D
cPLA_2_: Cytosolic PLA_2_
sPLA_2_: Secretory PLA_2_
LDL: Low-density lipoproteins
oxLDL: Oxidized LDL
MAPK: Mitogen-activated protein kinases
PE: Phosphatidyl ethanol
PA: Phosphatidic acid
MTT: 2-butanol, 3-(4,5-dimethylthiazol-2-yl)-2,5-diphenyl tetrazolium bromide
AA: Arachidonic acid
PTX: Pertussis toxin
MAFP: Methyl arachidonyl fluorophosphonate.
